# Novel strategies for expansion of tooth epithelial stem cells and ameloblast generation

**DOI:** 10.1038/s41598-020-60708-w

**Published:** 2020-03-18

**Authors:** Martin Binder, Leah C. Biggs, Mark S. Kronenberg, Pascal Schneider, Irma Thesleff, Anamaria Balic

**Affiliations:** 1 Research Program in Developmental Biology, Institute of Biotechnology, University of Helsinki, Helsinki, Finland; 2 Helsinki Institute of Life Science, Biomedicum Helsinki, University of Helsinki, Helsinki, Finland; 3 Wihuri Research Institute, Biomedicum Helsinki, University of Helsinki, Helsinki, Finland; 4 Stem Cells and Metabolism Research Program, Faculty of Medicine, University of Helsinki, Helsinki, Finland; 5 Center for Regenerative Medicine and Skeletal Development, School of Dental Medicine, UConn Health, Farmington, CT USA; 6 Department of Reconstructive Sciences, School of Dental Medicine, UConn Health, Farmington, CT USA; 7 Department of Biochemistry, University of Lausanne, CH-1066 Epalinges, Switzerland

**Keywords:** Stem cells, Stem-cell niche

## Abstract

Enamel is secreted by ameloblasts derived from tooth epithelial stem cells (SCs). Humans cannot repair or regenerate enamel, due to early loss of tooth epithelial SCs. Contrarily in the mouse incisors, epithelial SCs are maintained throughout life and endlessly generate ameloblasts, and thus enamel. Here we isolated Sox2-GFP+ tooth epithelial SCs which generated highly cellular spheres following a novel *in vitro* strategy. This system enabled analysis of SC regulation by various signaling molecules, and supported the stimulatory and inhibitory roles of Shh and Bmp, respectively; providing better insight into the heterogeneity of the SCs. Further, we generated a novel mouse reporter, *Enamelin*-tdTomato for identification of ameloblasts in live tissues and cells, and used it to demonstrate presence of ameloblasts in the new 3D co-culture system of dental SCs. Collectively, our results provide means of generating 3D tooth epithelium from adult SCs which can be utilized toward future generation of enamel.

## Introduction

Postnatally, stem cells (SC) remain within organ-specific SC niches and provide the source of reparative and regenerative potential. Continuously-growing rodent incisors contain epithelial and mesenchymal SC niches, which unceasingly produce the mineralized constituents of the adult tooth: enamel and dentin, respectively (reviewed in^1^). The epithelial SCs reside in the labial cervical loops (LaCL) situated at the proximal end of the tooth, and are surrounded by their progeny, as well as the mesenchymal cells of dental pulp and follicle. Within the LaCL, these SCs are located in the loosely-arranged stellate reticulum and can be identified as slowly dividing, label-retaining cells, which express Sox2, *Lgr5, Ptch1, Gli1, ABCG2, Bmi-1*, and *Oct3/4*, among other genes^2–7^. *In vivo* studies and *in vitro* explant cultures have demonstrated that the survival, proliferation, and differentiation of tooth epithelial SCs are regulated through complex, but not fully elucidated, interactions with the surrounding cells.

An intricate signaling network involving several signaling pathways, including FGF, BMP, Activin, and Follistatin, regulates maintenance of SCs, transit-amplifying cell (TAC) proliferation, and ameloblast differentiation (reviewed in^8^). In addition, we recently showed that functionally distinct Patched receptors establish multimodal Hedgehog signaling in the cervical loop^9^, which enables simultaneous regulation of maintenance of SCs and ameloblast differentiation, as previously reported^7,10^. Published data have demonstrated that Fgf10 and Fgf3 positively regulate SCs, similar to Shh signaling, while BMP4 and mesenchyme-derived Wnt signaling have a negative effect on this population^10–12^. Intriguingly, the incisor SC niche is characterized by exclusive lack of Wnt signaling, attributed to the enrichment of Wnt inhibitors^13^.

Epithelial SCs in mouse incisors continuously generate ameloblasts in a stepwise process during which the cells transit through increasing degrees of differentiation marked by the expression of specific molecular markers. The earliest progeny of epithelial SCs is characterized by increased expression of secreted frizzled-related protein 5 (*Sfrp5)*. The Sfrp5+ cells become transit-amplifying cells (TACs) which simultaneously downregulate the expression of *E-cadherin* and upregulate the expression of *P-cadherin*^5,14^. TACs also express nuclear Yap and TAZ and at their final stages acquire Sonic hedgehog (*Shh)* expression, which continues after their commitment to the ameloblast lineage^7,15,16^. Generation of pre-ameloblasts from Shh-expressing TACs is characterized by a downregulation of *P-cadherin* and reappearance of *E-cadherin* expression^14^. These cells further differentiate to secretory ameloblasts, expressing ameloblast-specific genes including *ameloblastin, amelogenin*, and *enamelin*, which are deposited in the enamel matrix^17–19^.

For many dental tissues, including dental pulp, *in vitro* cell culture systems have provided the means to gain insight into molecular regulation and cellular properties of their diverse cell populations, including SCs^20^. However, the ameloblast lineage has not yet been recapitulated in cell culture, and consequently, the enamel tissue has so far not been produced *in vitro* in cell culture. Enamel is a unique, mineralized tissue that is produced only once, during the development of the tooth crown, and the ability to regenerate enamel has been lost in humans. Recently, several studies reported protocols to culture epithelial cells isolated from the LaCL of mouse incisors in 3D *in vitro* using Matrigel^21–23^. These studies demonstrated generation of spherical structures of variable morphology, ranging from highly cellular spheres to non-specific, cyst-like morphology in which a layer of compact, polygonal cells surrounded an unspecified matrix core, suggestive of differentiation. These studies, however, have not demonstrated any evidence of enamel matrix secretion.

The current study was designed to develop a cell culture system which will enable *in vitro* expansion of tooth epithelial SC, as well as potentiate the development of an *in vitro* system capable of generating ameloblasts. Our results demonstrate that we can obtain tooth epithelial SCs by FACS using Sox2-GFP transgenic animals. Sox2-GFP+ cells represent a more homogenous population of postnatal SCs which we expanded *in vitro* using a non-adherent, sphere-forming assay supplemented with Shh protein. Importantly, our system enables analysis of the effect of various signaling molecules, as well as pharmacological compounds, on a homogenous tooth epithelial SC population. We also generated a novel transgenic reporter model, Enamelin-tdTomato, which enables identification of differentiated ameloblasts in live tissues. Furthermore, we have developed a novel protocol for co-culture of epithelial and mesenchymal adult SCs and demonstrated that it can be used to generate ameloblasts *in vitro*.

## Results

### Regulation of heterogeneous epithelial SCs in the cervical loops (CLs) by signaling molecules

**Figure 1:**
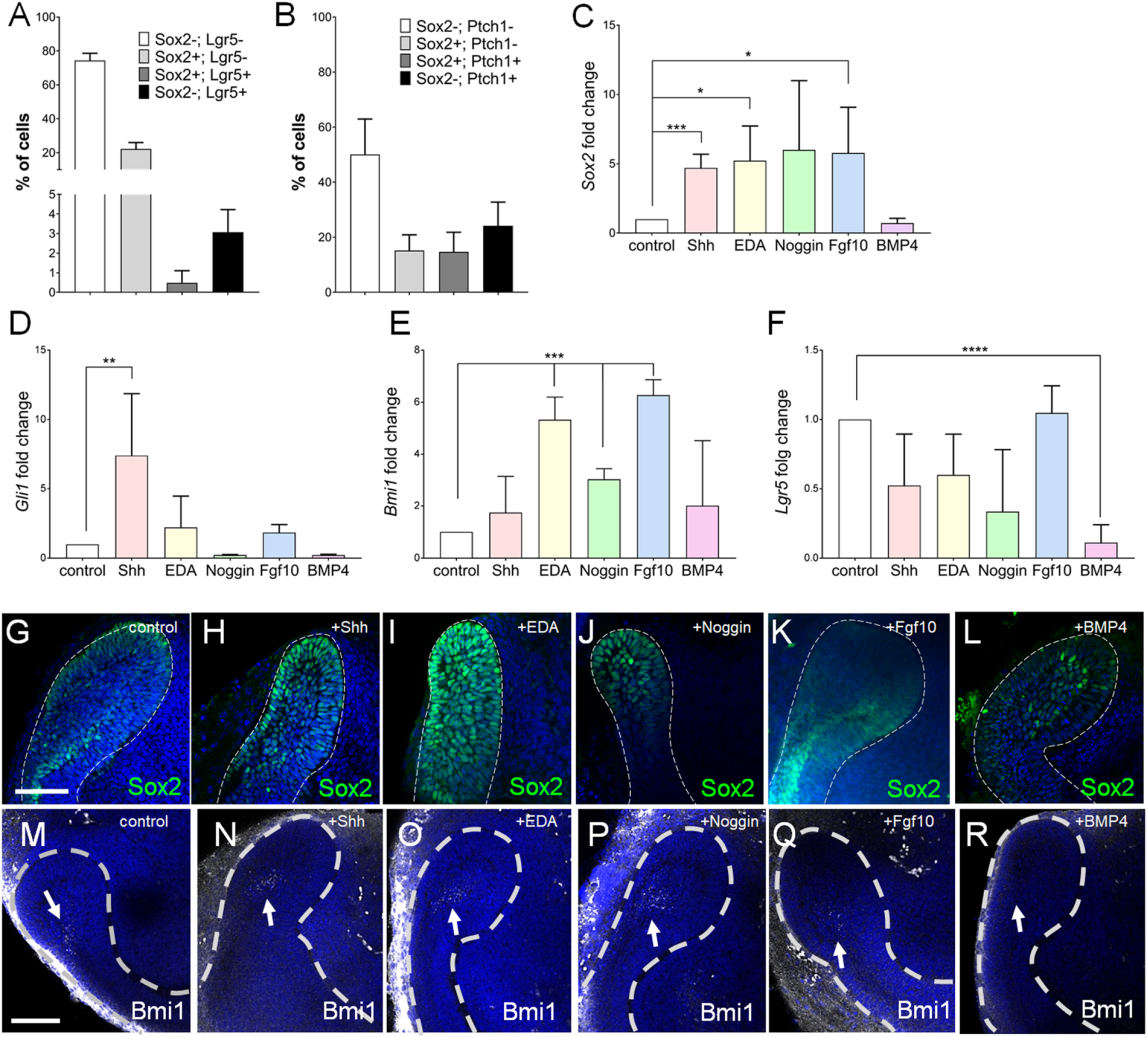
Regulation of Sox2-GFP+ cells by different signaling molecules. Quantification of flow cytometry analysis of Sox2-GFP and Lgr5 (**A**), and Sox2-GFP and Ptch1 expression (**B**) in the EpCAM+ epithelial compartment of the incisor. (**C**–**F**) RT-qPCR analysis of stem cell markers *Sox2* (C), *Gli1* (**D**), *Bmi1* (**E**) and *Lgr5* (**F**) in explants cultured 24 h with indicated proteins. N ≥ 3, *p value <0.03, **p value <0.01, ***p value <0.001, ****p value <0.0001 were determined by Student’s tTest or one-way ANOVA (*Gli1*). Immunostaining for Sox2 (**G**–**L**) and Bmi1 (**M**–**R**) in organ cultures of proximal ends of the P2 incisors cultured for 48 h with Shh (**H**,**N**), EDA (**I**,**O**), Noggin (**J**,**P**), Fgf10 (**K**,**Q**), and BMP4 (**L**,**R**). Nuclei were stained with DAPI. White dashed line outlines the cervical loop. Scale bar 100 μm.

SCs from various organs, including intestine, kidney and hair follicle have been successfully cultured in a 3 dimensional format in combination with addition of recombinant proteins, thus recapitulating the *in vivo* molecular landscape of their SC niche^24,25^. In the incisor labial CLs, the SCs are defined by the expression of several genes, including Sox2, Bmi1, Lgr5, Gli1 and Ptch1^2,5–7,13^, whose distinct, yet somewhat shared, expression domains indicate the heterogeneity of SCs within the niche^1^. We used flow cytometry analysis to analyze expression of Lgr5 and Ptch1 in the LaCLs, and correlated it with Sox2-GFP, a reporter for the Sox2+ SC population^5^. Our results demonstrate that Sox2 is an abundant SC marker, while markers Lgr5 and Ptch1 comprise a significantly smaller population (Fig. 1A,B) within the niche. While some of the Lgr5+ cells also expressed Sox2-GFP, the majority were exclusively Lgr5+ (Fig. 1A,B). Likewise, not all Ptch1+ SCs expressed Sox2-GFP (Fig. 1C,D).

Molecular heterogeneity of the Sox2+ SC population implies potential differences in the regulation of each subset, while some signaling pathways have been shown to regulate the whole SC niche. One of the major positive regulators of the SC niche is Fgf10, lack of which leads to depletion of the SC niche^11,26^. Furthermore, Shh-BMP4 antagonistic signaling axis has been shown to regulate maintenance of the Sox2+ SCs. We therefore used *in vitro* organ culture to screen the effect of these, and other molecules on various SC populations. We first analyzed their effects on the mRNA expression of several genes associated with SCs, including *Bmi1, Sox2, Gli1* and *Lgr5*. Our results demonstrate that Shh regulated mainly *Sox2* and *Gli1* expression (Fig. 1C,D), EDA and Fgf10 positively regulated *Sox2* and *Bmi1* (Fig. 1C,E), while Noggin positively regulated *Bmi1* expression (Fig. 1E). Interestingly, expression of *Lgr5* was regulated only by Bmp4, in a negative manner (Fig. 1F). These results were further corroborated by analysis of the protein expression. We observed an increase in the number of Sox2+ cells in explants treated with Shh and EDA only (Fig. 1H,I), although Sox2-GFP expression was upregulated in presence of all signaling molecules, except Bmp4 (Fig S1B–G). Number of Bmi1+ cells was increased in explants treated with Shh, EDA and Noggin (white arrows in Fig. 1O,P), while Lgr5 expression domain expanded in explants treated with Fgf10 and EDA (Fig S1L,J). Collectively, these data demonstrate that a molecular heterogeneity is accompanied by differential regulation of tooth epithelial SC population.

### Sox2**+** dental SCs from epithelial cervical loops can be expanded in sphere cultures

Previous studies demonstrated that epithelial cells isolated from the LaCL of mouse incisors and cultured *in vitro* within Matrigel form 3D organoids composed of a layer of compact, polygonal cells surrounding an unspecified matrix core^21–23^. This suggests that the majority of the cells within organoids had started their differentiation program, most likely induced by Matrigel components, which include collagen type I that supports ameloblast differentiation^27–29^. We aimed at developing an *in vitro* system which would maintain tooth epithelial SCs in an undifferentiated state and enable expansion of their numbers, while preventing their commitment and differentiation.

**Figure 2:**
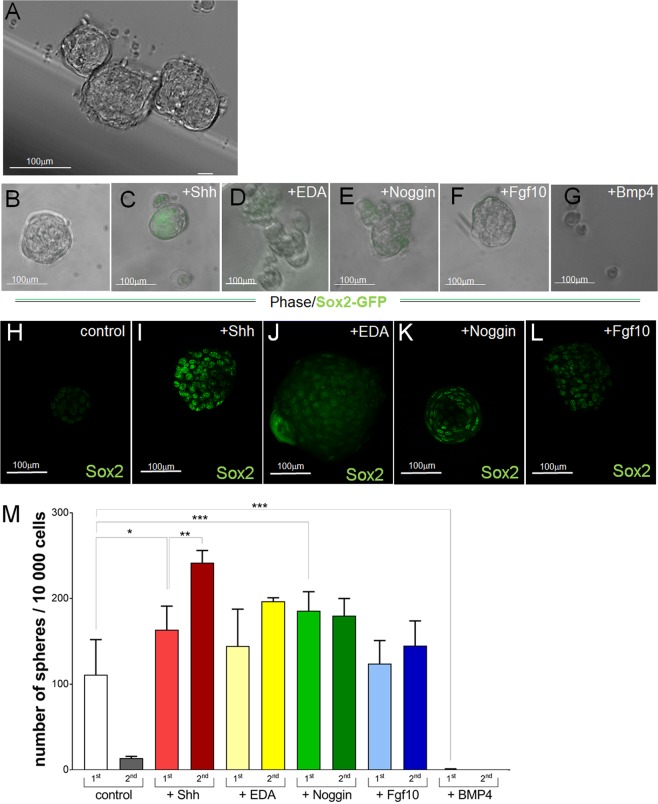
*In vitro* culture of epithelial cells isolated from cervical loops. (**A**) Spheres formed in non-adherent cultures of epithelial cells isolated from cervical loops over the course of 14 days. Analysis of Sox2-GFP expression (**B**–**G**) and Sox2 protein expression in non-adherent cultures of the whole epithelium after 14 days of culture. (**M**) Comparison of sphere formation capacity after the first (1^st^) and second (2^nd^) passages (number of spheres formed per 10 000 cells). N ≥ 4, *p value <0.025, **p value <0.01 and ***p value <0.001 were determined by Mann-Whitney test. Scale bar 100 μm.

A single cell suspension of epithelial cells obtained from the incisor LaCLs of Sox2-GFP mice was cultured under non-adherent conditions. Over the course of 14 days the cells formed spheres of variable size (Fig. 2A), including bigger spheres which resembled the previously reported organoids (Fig. S2A), and highly cellular, smaller spheres (Fig. S2B). The smaller spheres contained few cells expressing Sox2-GFP, as well as cells expressing TAC marker^14^ P-cadherin (Fig. S2A,C). Sox2-GFP expression in these spheres indicates presence of SCs with self-renewal ability, which was tested by assaying the sphere-forming capacity after repeated enzymatic dispersion and passaging of the spheres (Fig. S2D–F). We found that the number of spheres dramatically decreased after the first passage (Fig. S2G), which was most likely due to a loss of Sox2-GFP+ SCs. Indeed, not all cells expressed GFP in the initial cultures (Fig. S2B,H), and the expression of Sox2-GFP by day 14 was almost undetectable (Fig. 2B).

Addition of Shh maintained Sox2-GFP during the entire culture period (Fig. 2C) which correlated with increased number of spheres formed through passaging, as compared to control (Fig. 2M). Addition of other signaling proteins had little (EDA, Noggin) or no (Fgf10, BMP4) effect on Sox2-GFP expression (Fig. 2D–G), or on the expression of endogenous Sox2 protein (Fig. 2J–L). Conversely, addition of EDA and Fgf10 had no effect on the sphere formation capacity after passaging (Fig. 2M). Surprisingly, Noggin increased sphere formation in the first passage, but had no effect on the sphere formation afterward (Fig. 2M). Expectedly, BMP4 completely abrogated the sphere-forming capacity (Fig. 2M). These data show that addition of Shh alone significantly increased the number of spheres from passage 1 to passage 2. In these cultures 50% increase in the sphere number between the passages (from 163 ± 26 to 242 ± 11 spheres/10 000 cells) suggests that the SCs and the sphere-forming ability are maintained for at least one passage more, as compared to control and cultures treated with other proteins tested in our study.

### *In vitro* sphere formation assay can be used to efficiently culture and expand Sox2-GFP**+** SCs

**Figure 3:**
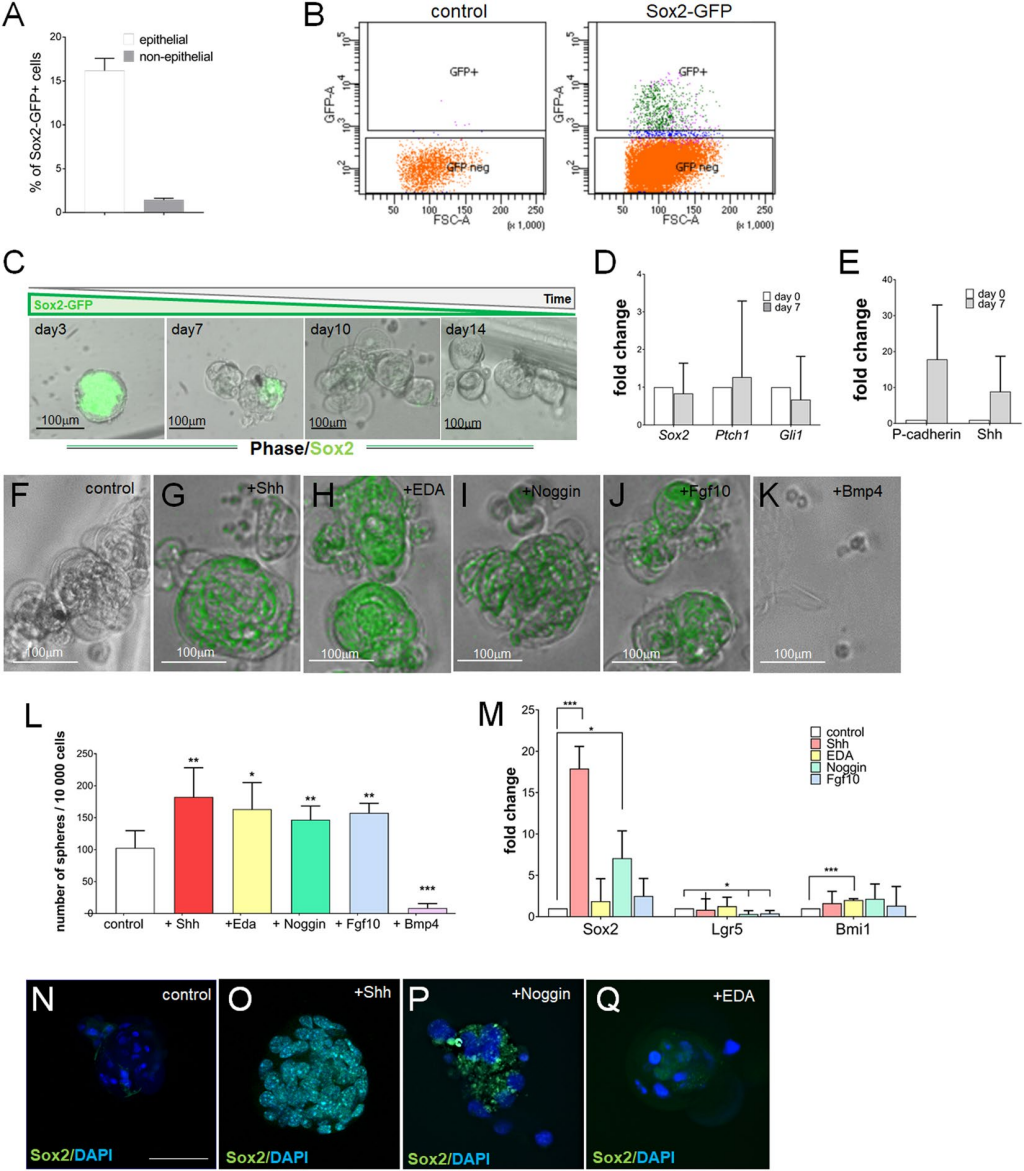
Isolation and *in vitro* culture of Sox2-GFP+ stem cells. (**A**) Quantification of flow cytometry analysis of Sox2-GFP expression in the cells obtained from proximal ends of the incisor. (**B**) Strategy for FACS sorting of LaCL cells derived from Sox2-GFP+ animals. (**C**) Temporal analysis of Sox2-GFP expression in control cultures. (**D**) RT-qPCR analysis of stem cell markers *Sox2, Ptch1* and *Gli1* at day 7 of culture. (**E**) RT-qPCR analysis of TAC markers *P-cadherin* and *Shh* at day 7 of culture. (**F**–**K**) Analysis of Sox2-GFP expression in spheres after 14 days of culture. (**L**) Sphere formation capacity (number of spheres formed per 10 000 cells). N ≥ 4, *p value < 0.025, **p value < 0.01 and ***p value < 0.001 were determined by Mann-Whitney test. (**M**) RT-qPCR analysis of stem cell markers *Sox2, Lgr5* and *Bmi1* in 14 days old cultures. N = 3, *p value < 0.05 and ***p value < 0.001 were determined by Student’s tTest. (**N**–**Q**) Immunostaining for Sox2 protein in 14 days old spheres. Nuclei are stained with DAPI. Scale bar 100 μm.

We demonstrated that the Sox2-GFP+ population (hereafter referred to as GFP+ cells) represents 20–25% of the cervical loop niche population (Fig. 1A). Given the published role of Sox2 in the tooth epithelial stem cells^5^, we have decided to enrich for the Sox2 expressing cells using FACS sorting. Approximately 500–1000 GFP+ cells were obtained from each Sox2-GFP transgenic mouse (Fig. 3B). During initial days under non-adherent culture conditions, the majority of the cells within each sphere expressed GFP at high levels, but the number of GFP+ cells and the intensity of GFP steadily decreased (Fig. 3C). GFP+ cells were lost between days 7 and 10, at which time cells expressing *P-cadherin* and *Shh* were present in the spheres (Fig. 3E), which indicated that the Sox2-GFP+ SC populations commenced their differentiation into TACs. Individual addition of Shh, EDA, Noggin or Fgf10 during the first 7 days of culture maintained high levels of GFP expression during the entire culture period (Fig. 3F–K) and had a positive effect on the sphere-formation capacity (Fig. 3L). However, only cultures treated with Shh and Noggin demonstrated increased *Sox2* expression, with no increase in the expression of other SC markers such as *Lgr5* and *Bmi1*, but surprisingly inhibited *Lgr5* expression in spheres treated with Noggin (Fig. 3M). Interestingly, co-administration of Shh and Noggin had a positive effect on both sphere-formation capacity (Fig. S3A,B) and *Sox2* expression (Fig. S3C), but the changes were comparable to those observed in cultures treated with Shh only. Furthermore, cultures treated with Shh-Noggin combination contained cells expressing TAC markers *P-cadherin* and *Shh*, at levels higher than those detected in cultures treated with Shh only (Fig. S3D). Bmp4, as expected, exhibited a negative effect on sphere formation (Fig. 3G,H), as well as on *Sox2* expression (Fig. 3H). Interestingly, EDA treatment increased *Bmi1* expression in the spheres, which recapitulated its effect on this SC marker in explant culture (Fig. 3M).

### Generation of ameloblast reporter model

We next tested whether our *in vitro* sphere forming assay could be utilized for generation of ameloblasts from the adult Sox2+ tooth epithelial SCs. Presence of *P-cadherin* and *Shh* expression at day 7 (Fig. 3E) indicated commitment and differentiation of SCs into TACs that generate ameloblasts. Previous studies have indicated that Shh signaling is crucial for differentiation of TACs into ameloblasts^7^. The multimodal Shh signaling which enables simultaneous regulation of SC maintenance and differentiation of TACs to ameloblasts has been attributed to the functionally distinct Ptch receptors^30^. We hypothesized that addition of Shh to spheres which contained TACs might induce ameloblast differentiation, and therefore supplemented the culture media of untreated spheres with Shh, from day 7 of culture. Complete loss of Sox2-GFP expression in the following week indicated that the differentiation of Sox2+ SCs continued (Fig. S4B), which was further supported by a decrease in *P-cadherin* expression (Fig. S4G). However, low to undetectable levels of *Enamelin*, a molecular marker of ameloblasts, suggested absence of ameloblasts in these spheres.

**Figure 4:**
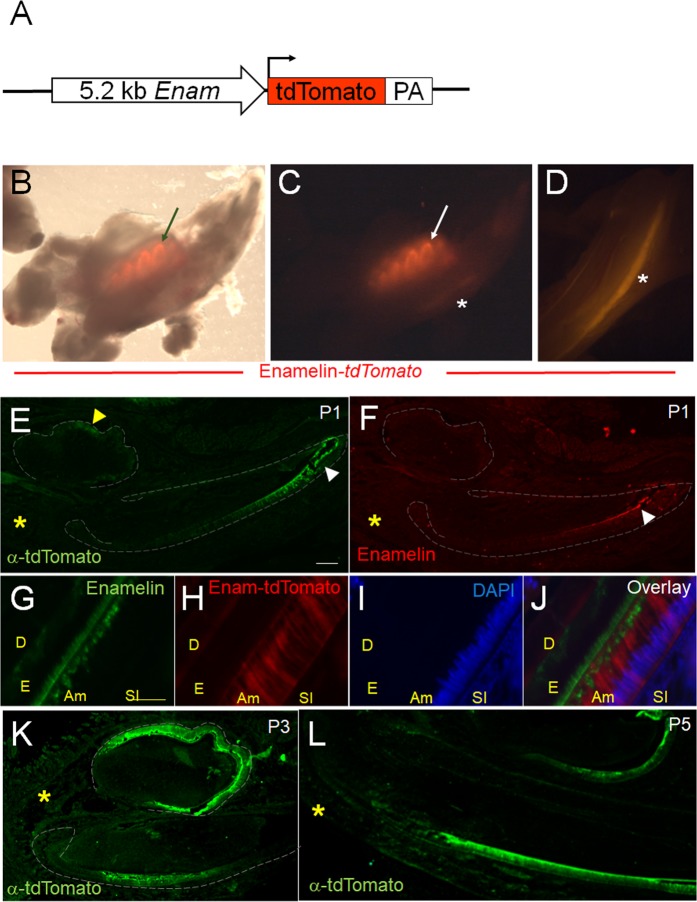
Enamelin-tdTomato transgenic reporter identifies ameloblasts *in vivo*. (**A**) Phase contrast-fluorescent light overlay of one side of the mandible isolated from 5 days old Enamelin-tdT transgenic mouse demonstrates red fluorescence in the molar crowns (arrow). (**B**,**C**) Fluorescent light images of the mandible from (**B**) showing red fluorescent molar crowns (arrow) and at the labial side of the incisor (asterisk). (**E**–**L**) Correlation of tdTomato and endogenous Enamelin protein expression in mandibles of 1 day (**E**–**J**), 3 day (**K**) and 5 day (**L**) old mice. tdTomato was analyzed by immunolabeling (**E**,**K**,**L**) or by confocal imaging of the endogenous fluorescent protein (**G**). Scale bar 100 μm. Yellow scale bar 20 μm. Abbreviations: D = dentin, E = enamel, Am = ameloblast, SI = stratum intermedium.

In order to facilitate identification of ameloblasts in live cells we generated a novel mouse reporter, in which tdTomato fluorescent protein (tdT) is driven by a 5.1-kilobase long Enamelin promoter fragment (Fig. 4A) that drives gene expression specifically in ameloblasts^31^. At P5, red fluorescence outlined the crowns of the developing molars (arrow in Fig. 4B,C), as well as the labial side of the developing incisors (asterisk in Fig. 4C,D). Expression of tdT was observed as early as P1 in the cells located at the tips of the growing incisor (white arrowhead in Fig. 4E) and molar cusps (yellow arrowhead in Fig. 4F) and it co-localized with the endogenous Enamelin protein (Fig. 4F,G–J) expressed by the secretory ameloblasts. In the following days, expression of the fluorescent reporter expanded and was detected in the secretory ameloblasts, in the growing incisors, and the entire layer of ameloblasts covering molar crowns (Fig. 4K,L). As expected, no transgene expression was detected on the lingual side of the incisor, or in the odontoblasts and the surrounding bone and soft tissues (yellow asterisk in Fig. 4). These data indicated that *Enamelin*-tdT is an exclusive reporter for differentiated ameloblasts and can be used for their identification *in vivo*.

### 3D co-culture system of adult epithelial and mesenchymal stem/progenitor cells

**Figure 5:**
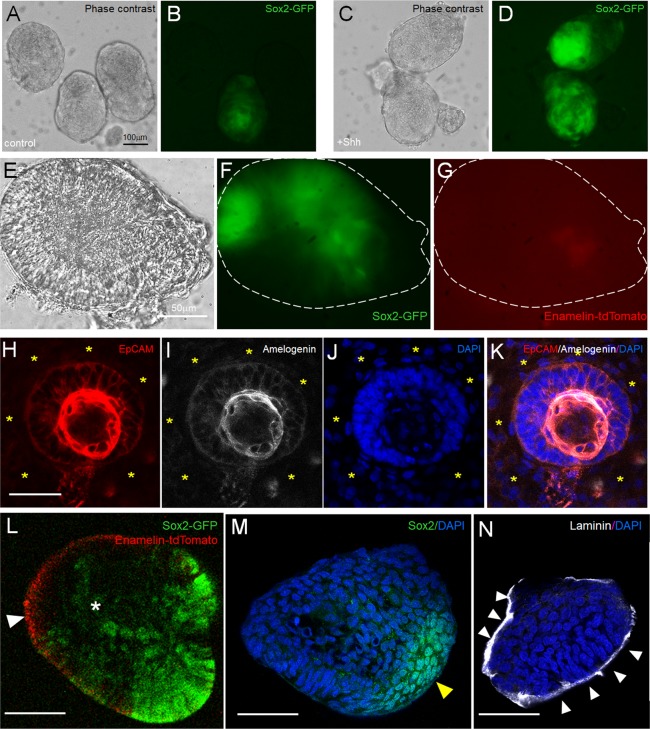
3D *in vitro* co-culture of epithelial and mesenchymal stem cells. (**A**–**D**) Spheres from control (**A**,**B**) and Shh treated (**C**,**D**) co-cultures at day 5 imaged under phase contrast (**A**,**C**) and fluorescent light (**B**,**D**) to detect Sox2-GFP expression. Phase contrast (**E**), Sox2-GFP (**F**) and Enamelin-tdTomato (**G**) expression in Shh-treated sphere after 3 days in Matrigel. (**H**–**K**) Immunostaining for EpCAM (**H**) and Amelogenin (**I**) in the Shh treated sphere after 3 days in Matrigel. Nuclei were stained by DAPI (**J**). (**L**) Shh-treated sphere live imaged under confocal microscope after 3 days in Matrigel. White arrowhead points to Enamelin-tdTomato expression, and yellow arrowhead indicates elongated cells expressing Sox2-GFP. (**M**,**N**) Immunostaining for Sox2 (**M**) and Laminin (**N**) in adjacent cryosections of the sphere from (**N**). Scale bar 100 μm (black) and 50 μm (white).

Based on published data, ameloblast differentiation occurs only in the presence of the dental mesenchyme (reviewed in^8^). We next aimed at developing a 3D culture system which will permit the epithelial-mesenchymal interactions, and could be utilized toward achieving amelogenesis *in vitro*. We dissected the proximal end of the incisor, and separated the entire cervical loop together with the adjacent mesenchyme from Sox2-GFP+; Enamelin-TdTomato+ mice (encircled area in Fig. S5A). The obtained samples, which did not contain Enamelin-tdTomato+ cells, and the expression of *Ameloblastin, Amelogenin* and *Enamelin* was low to undetectable (Fig. S5B), were enzymatically dispersed and the single cell suspension was cultured under the non-adherent conditions in high density for 5 days. During this time the cells generated spheres which contained both GFP+ and GFP- cells (Fig. 5B). Addition of Shh increased the number of GFP+ cells and the intensity of GFP (Fig. 5D). In addition, the mesenchymal and epithelial compartments could clearly be distinguished within the spheres (Fig. S5C). The spheres were collected at day 5 and placed in Matrigel. Within the following three days epithelial cells from Shh-treated cultures had generated 3D structures lined by elongated cells, of which some expressed high levels of Sox2-GFP (Fig. 5E,F). Only faint levels of Enamelin-tdTomato were detected in these structures (Fig. 5G). The majority of the mesenchymal cells have migrated out of the spheres and into the Matrigel, while only few remained within the sphere, as indicated by Vimentin immunostaining and *Col1a1* expression (Fig. S5D,E). *In situ* hybridization for *Ameloblastin* on sectioned spheres demonstrated that some cells have started ameloblast differentiation (Fig. S5F). While we detected Amelogenin-producing cells in the epithelial compartment of the 3D cultures (Fig. 5H–K), live confocal imaging demonstrated faint levels of Enamelin-tdTomato (white arrowhead in Fig. 5L), suggesting that the cells which express Amelogenin are most likely preameloblasts. Interestingly, Sox2 expressing cells (identified by GFP, Fig. 5L, and immunostaining using anti-Sox2 antibody, Fig. 5M) were detected in a localized, rather than randomly assorted, manner within the spheres, reminiscent of the LaCL. In addition, a band of laminin staining suggested generation of basement membrane around the epithelial cells (white arrowheads in Fig. 5N).

## Discussion

In this study the restricted subpopulation of adult tooth epithelial SCs was successfully isolated and expanded *in vitro*. Our results also demonstrate that the non-adherent sphere-forming assay provides a suitable platform to analyze the effects of various signaling molecules and pharmacological compounds on tooth epithelial cells at various stages of differentiation, including SCs. We also show that this system can be used for co-culture of adult tooth stem cells, both mesenchymal and epithelial.

The epithelial subset which contains the SC population in the mouse incisors is molecularly heterogeneous, as suggested by distinct, yet somewhat overlapping expression domains of various genes (reviewed in^1^), including Sox2, Lgr5, and Ptch1 analyzed in this study. The observed molecular heterogeneity may indicate the presence of several SC populations which each generate one or more tooth epithelial lineages; alternatively, the diverse molecular pattern may indicate a variety of stemness states of a single SC progeny. Our flow cytometry analysis demonstrated the diversity of Sox2-GFP+ SC population, in which some cells also express Ptch1, or, as recently reported, Lgr5^32^.

Molecular heterogeneity of SCs also suggests differences in the regulation of this heterogeneous population, which was further supported by our analysis of the effects of various signaling molecules on this population. Previous studies have shown that an antagonistic Shh-BMP signaling axis maintains the SC population in cervical loops^10^, and in this study we also demonstrated opposite effects of Shh and BMP4 on the propagation and maintenance of the Sox2-GFP+ SCs. In our *in vitro* system, however, inhibition of BMP signaling by Noggin had a milder effect on the sphere formation and *Sox2* expression, suggesting that Hedgehog signaling is the key regulator of SCs. This is in line with our recent study which indicated that Hedgehog signaling transduced through Ptch1 receptor is a main regulator of Sox2 expressing SCs^9^. BMP4 signaling in the incisor cervical loop induces ameloblast differentiation^33^ and negatively regulates the tooth epithelial SC niche^10^. Here, we showed in explant culture that the negative effect of BMP4 on SCs most likely involves inhibition of Lgr5 mRNA and protein expression. The Lgr5 population is critical for the maintenance of the SC niche and its loss is associated with the loss of Sox2+ SCs and a decrease in the size of the CL^12^. These data collectively demonstrate that our *in vitro* system can be used to analyze the direct effect of many signaling molecules on the small subset of tooth epithelial stem cells. Though this system is a critical advancement in the study of tooth epithelial stem cells, it is not yet suitable to evaluate SC self-renewal.

*In vitro* cell cultures and fluorescent reporters have played crucial roles for better understanding of cellular heterogeneity in the tooth mesenchyme (dental pulp)^20^. Despite considerable efforts, an efficient protocol enabling primary cultures of the tooth epithelial SCs is lacking. Unlike the previous studies in which epithelial cells from LaCL generated nonspecific organoids^21–23^, we have developed a culture system which enables isolation and propagation of a homogeneous population of Sox2-GFP+ SCs. This *in vitro* system provided the means to culture low numbers of cells, and yet generate highly cellular spheres which provide a source of 3D tooth epithelium that can be utilized in tissue recombination studies. Most importantly, our system provides necessary steps toward generation of enamel, which has so far not been produced *in vitro*, from SCs. These steps include generation of Enamelin-tdT transgenic reporter mouse model which enables ameloblast identification in live tissues, and can potentially be used for their isolation and subsequent molecular analyses at unprecedented resolution. Further, the co-culture studies will likely provide the foundation for the generation of enamel *in vitro*. The 3D nature of the co-cultures offers a physiological format for the epithelial-mesenchymal interactions, and generation of properly patterned tooth matrices, including tubular dentin and enamel, neither of which has yet been produced from stem cells *in vitro*.

### Experimental procedures

#### Animals

In this study we used previously described Sox2-GFP reporter mice^34^, and newly generated Enamelin-tdTomato reporter mice. In the latter model, a 5.2 kb fragment of the Enamelin promoter^35^ drives the expression of tdTomato protein. The tdTomato cDNA was inserted between *XbaI and XhoI* sites and the enamelin–tdTomato DNA construct was released from the vector by restriction endonuclease digestion with *KpnI* and *XhoI* and purified using a Qiaquick gel extraction kit (Qiagen, Valencia, CA, USA). Each purified construct was microinjected into FVB-derived oocytes to generate, in total, three productive transgenic founders. All work was performed at the GM Unit of the Animal Facility at the University of Helsinki. All three lines used for subsequent studies were maintained as hemizygotes. Tail DNA was isolated and genotyped by PCR using FWD primer: 5′-GCGAGGAGGTCATCAAAGAG-3′ and REV primer: 5′-CCGTCCTCGAAGTTCATCAC-3′, which bind within the tdTomato sequence. All experimental procedures in this study involving mice were approved by Ethical Committees on the Use and Care of Animals and the Animal Facility at the University of Helsinki.

#### Tissue isolation and culture

The proximal ends of the incisors were dissected from lower jaws of 2–3 weeks old postnatal mice in Dulbecco’s phosphate buffered saline (PBS), pH 7.4 and cultured in Trowell-type organ culture as previously described^36^. Cultures were treated with 100ng/ml of Shh, EDA, Fgf10, Noggin or BMP4, and media was changed every other day.

#### Cell cultures

Incisor proximal ends were dissected from 2–3 weeks old postnatal mice and LaCLs were manually separated from the surrounding mesenchyme. Isolated LaCLs were digested with 2 U/ml of Collagenase P (Roche, USA) in phosphate buffered saline (PBS) at 37 °C for 30–45 min on a rocking platform. Single-cell suspensions were either immediately cultured or FACS sorted based on GFP expression using FACSAria II or BD Influx flow cytometers with a 130-μm nozzle. Cells obtained were cultured in ultra-low attachment plates, at the density of 10 000 cells/ml of media composed of DMEM:F12 (Thermo Fisher), 100 U/ml Pen-Strep, B-27 Supplement (Thermo Fisher Scientific, USA), 20 ng/ml of bFGF, and 20 ng/ml of EGF, as previously described^37^. Cultures were treated with Noggin (R&D Systems, USA), Fgf10 (Peprotech, Sweden), Shh (R&D Systems, USA), EDA1^38^ and BMP4 (R&D Systems, USA) in a concentration 100 ng/ml of media. Media was refreshed every 3 days during the entire culture period (up to 14 days).

LaCLs with the most adjacent mesenchyme were dissected from mice carrying both Sox2-GFP and Enamelin-tdTomato transgenes and enzymatically dispersed to a single cell suspension. Cell were cultured at high density of 25 000 cells/ml as described earlier. On day 5 the spheres were collected, mixed with Matrigel and cultured for another 3 days in the presence of the media used for non-adherent cultures described earlier in the text.

#### Flow cytometry analysis

Proximal ends of the incisors were isolated and enzymatically processed to single cell solution. For the analysis, approximately 0.5–1 × 10^6^ cells was stained with pre-titrated antibodies, washed and resuspended in 300μl of media. Antibodies used were CD326 (EpCAM, 1:1000, BV711, BD Horizon 563134) and either Ptch1 (1:100, Santa Cruz sc-6149) or Lgr5 (1:250, Santa Cruz sc-68580) and secondary antibodies (AF568 or AF647, Invitrogen Molecular Probes, 1:500). 10,000–50,000 cells was analysed on LSRFortessa Analyzer.

#### Immunostaining

Immunostaining was conducted on whole explants following the established protocol^12^, or on spheres. Collected spheres were fixed with 1% Formalin for 30 min at room temperature, permeabilized in 0.5% Triton X-100 in PBS and incubated with blocking solution (10% normal goat serum, 1% BSA, 0.5% Triton in PBS) for 1 h at room temperature. Samples were then incubated with primary antibodies, followed by incubation with secondary antibodies.

Cryosections were fixed on the slide using 4% paraformaldehyde for 10 min at room temperature. Sections were then washed with PBS and incubated with blocking solution (10% normal goat serum, 1% BSA, 0.1% Triton in PBS) for 1 h at room temperature. Incubation with primary antibodies was performed for 2 h at room temperature, followed by incubation with secondary antibodies for 45 min at room temperature. Sections were washed and mounted using Vectashield Antifade Mounting medium with DAPI (Vector Laboratories, Burlingame, California, US).

Antibodies used are as follows: Sox2 (1:250, goat: Santa Cruz sc-17320), Bmi1 (1:250, Invitrogen, PA529891), EpCAM (1:1000, BD Pharmigen, 552370), Vimentin (1:400, Santa Cruz, sc-7557-R), Laminin (1:500, Sigma, L9393), Amelogenin (1:100, kind gift from Dr. Yasuo Yamakoshi, University of Michigan, USA) and Lgr5 (1:250, Santa Cruz sc-68580). Alexa Fluor conjugated secondary antibodies were used at 1:500 dilution and DAPI staining (1:2000) was used to label nuclei. Samples were visualized using a Zeiss LSM 700 confocal microscope and images analyzed with Zen 2012 (Carl Zeiss, Germany).

#### RNA Isolation and RT-qPCR

Spheres were collected after 14 days of culture and total RNA was isolated using RNeasy Plus Micro Kit (Qiagen), according to the manufacturer’s instructions, followed by cDNA synthesis using Quantitect Reverse Transcription Kit (Qiagen). Multiplex RT-qPCR was performed, enabling simultaneous detection of multiple targets in a single reaction well, utilizing a different probe for each target. All probes were calibrated on control cDNA to ensure that they can be distinguished from each other and simultaneously detect different targets. Each sample was analyzed in triplicate and data were normalized to GAPDH and Hprt expression. Probes used were all purchased from Bio-Rad and are as follows: Sox2 (10031225, qMmuCEP0060283), P-cadherin (10031231, qMmuCIP0030319), Gli1 (10031234, qMmuCEP0054131), Bmi1 (10031237, qMmuCEP0043063) and Lgr5 (10031234, qMmuCEP0053421).

#### Radioactive *in situ* hybridization

Spheres growing in Matrigel were fixed in 4% paraformaldehyde for 1 h at room temperature, dehydrated, paraffin embedded and sectioned at 5 μm thickness. Sections were processed for radioactive *in situ* hybridization following the standard protocol^39^. Expression of *Ameloblastin* and *Col1a1* was detected using [35S]-UTP (Perkin Elmer)-labeled RNA probes generated from plasmids kindly gifted by Dr. D’Souza (Univ. of Utah, USA) and Dr. Olsen (Harvard Medical School, USA), respectively.

#### Cryosectioning

Spheres growing in matrigel were collected and embedded in OCT media (Sakura Finetek, Tokyo, Japan) without fixation. The following day samples were sectioned using Leica CM3050 cryotome (Leica, Wetzlar, Germany) to 20 μm thick slices. Adjacent sections were processed for immunostaining.

#### Statistical analysis

Student’s T-test, one-way ANOVA or Mann-Whitney tests were used for statistical analysis, as indicated for each data set. The results are presented as mean ± SEM and *p* value <0.05 was used as the criterion for statistical significance.

### Ethical approval and informed consent

All experimental protocols we*re* approved by Ethical Committees on the Use and Care of Animals and the Animal Facility at the University of Helsinki. The methods were carried out in accordance with the relevant guidelines and regulations.

## Supplementary information

Supplementary Dataset 1.

## Data Availability

All data generated or analysed during this study are included in this published article (and its Supplementary Information files).
