# Supplementary material for: Novel strategies for expansion of tooth epithelial stem cells and ameloblast generation
Source: Sci Rep. 2020 Mar 18;10:4963. doi: 10.1038/s41598-020-60708-w (PMC7080756; doi:10.1038/s41598-020-60708-w)
Supplement: Supplementary file 1 — Supplementary Dataset 1. [file 41598_2020_60708_MOESM1_ESM.docx]

**Novel strategies for expansion of tooth epithelial stem cells and ameloblast generation**

Martin Binder^1^, Leah C. Biggs^2,3,4^, Mark S. Kronenberg^5,6^, Pascal Schneider^7^, Irma Thesleff^1^ and Anamaria Balic^1,^*****

Author Affiliations:

^1^Research Program in Developmental Biology, Institute of Biotechnology, University of

Helsinki, Helsinki, Finland.

^2^Helsinki Institute of Life Science, Biomedicum Helsinki, University of Helsinki, Finland

^3^Wihuri Research Institute, Biomedicum Helsinki, University of Helsinki, Finland

^4^Stem Cells and Metabolism Research Program, Faculty of Medicine, University of Helsinki,

Finland

^5^Center for Regenerative Medicine and Skeletal Development, School of Dental Medicine,

UConn Health, Farmington, CT, USA.

^6^Department of Reconstructive Sciences, School of Dental Medicine, UConn Health, Farmington, CT, USA.

^7^Department of Biochemistry, University of Lausanne, CH-1066 Epalinges, Switzerland.

*****Corresponding author. E-mail: [anamaria.balic@helsinki.fi](mailto:anamaria.balic@helsinki.fi)

**Supplemental Figures**


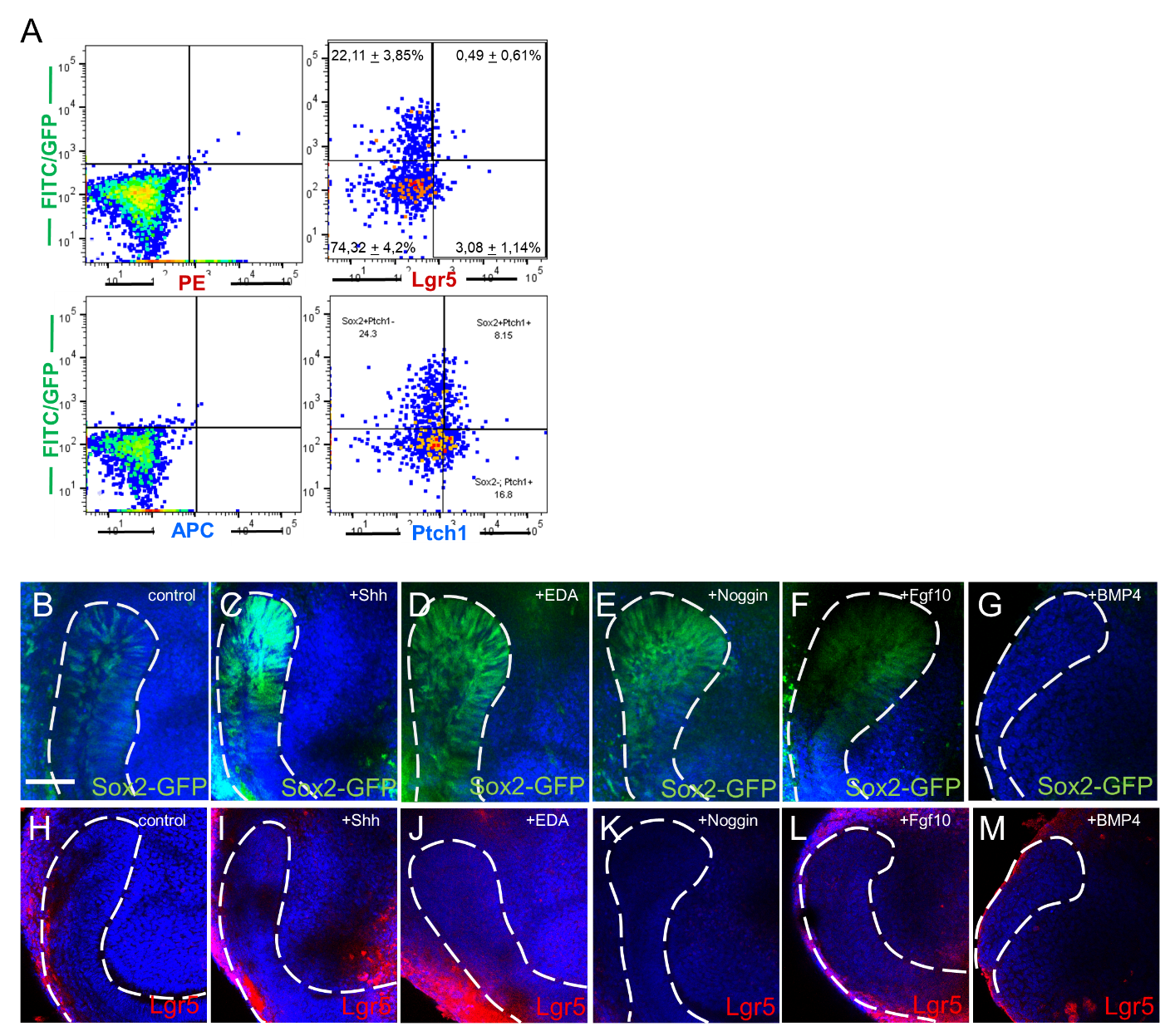


**Supplemental Figure 1. Regulation of epithelial stem cells in organ culture.** (A) Plots of flow cytometry analysis of Sox2-GFP expression in correlation with Lgr5 (upper row) and Ptch1 (bottom row) expression. Left side of the panel are negative controls stained with secondary antibody after incubation with goat serum. (B-G) Analysis of Sox2-GFP expression and immunostaining for Lgr5 (H-M) in cervical loops of explants of proximal ends of the P2 incisors cultured 48h with Shh, Noggin, Fgf10, EDA, or Bmp4, as indicated. Nuclei were stained with DAPI. White dashed line outlines the cervical loop. Scale bar 100 μm.
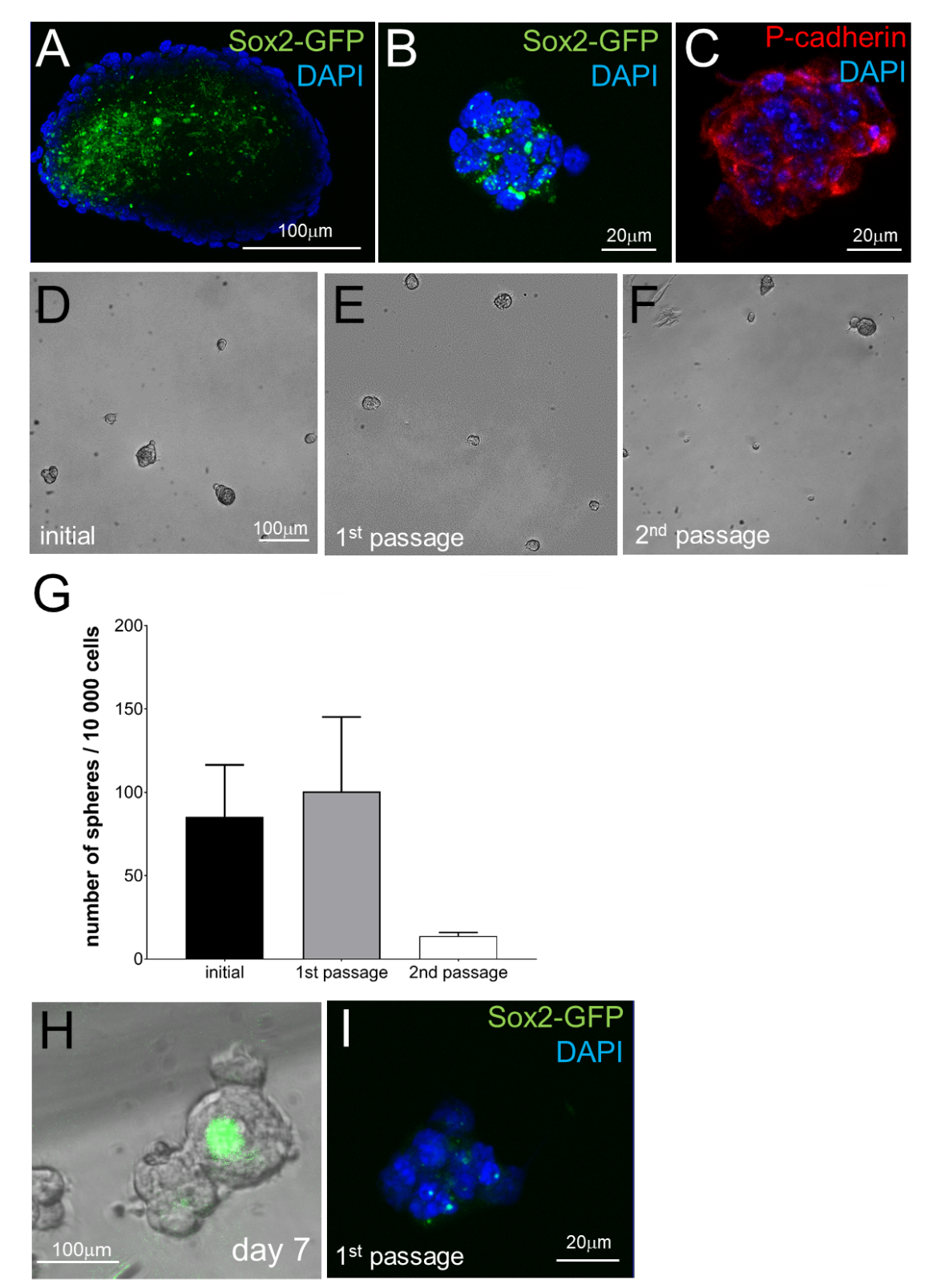


**Supplemental Figure 2. Sphere forming ability of epithelial cells from incisor cervical loops.** (A, B) Expression of endogenous Sox2-GFP in spheres resembling organoids (A) and highly cellular spheres (B). (C) Immunostaining for P-cadherin in the highly cellular spheres. (D) Spheres generated from epithelial cells from LaCLs after initial culture, and after the first (E) and second (F) passage. (G) Quantification of spheres formed after each passaging. (H) Sox2-GFP expression in the initial culture at day 7. (I) Immunostaining for Sox2 in the first passage sphere at day 7. Scale bar 100 μm.


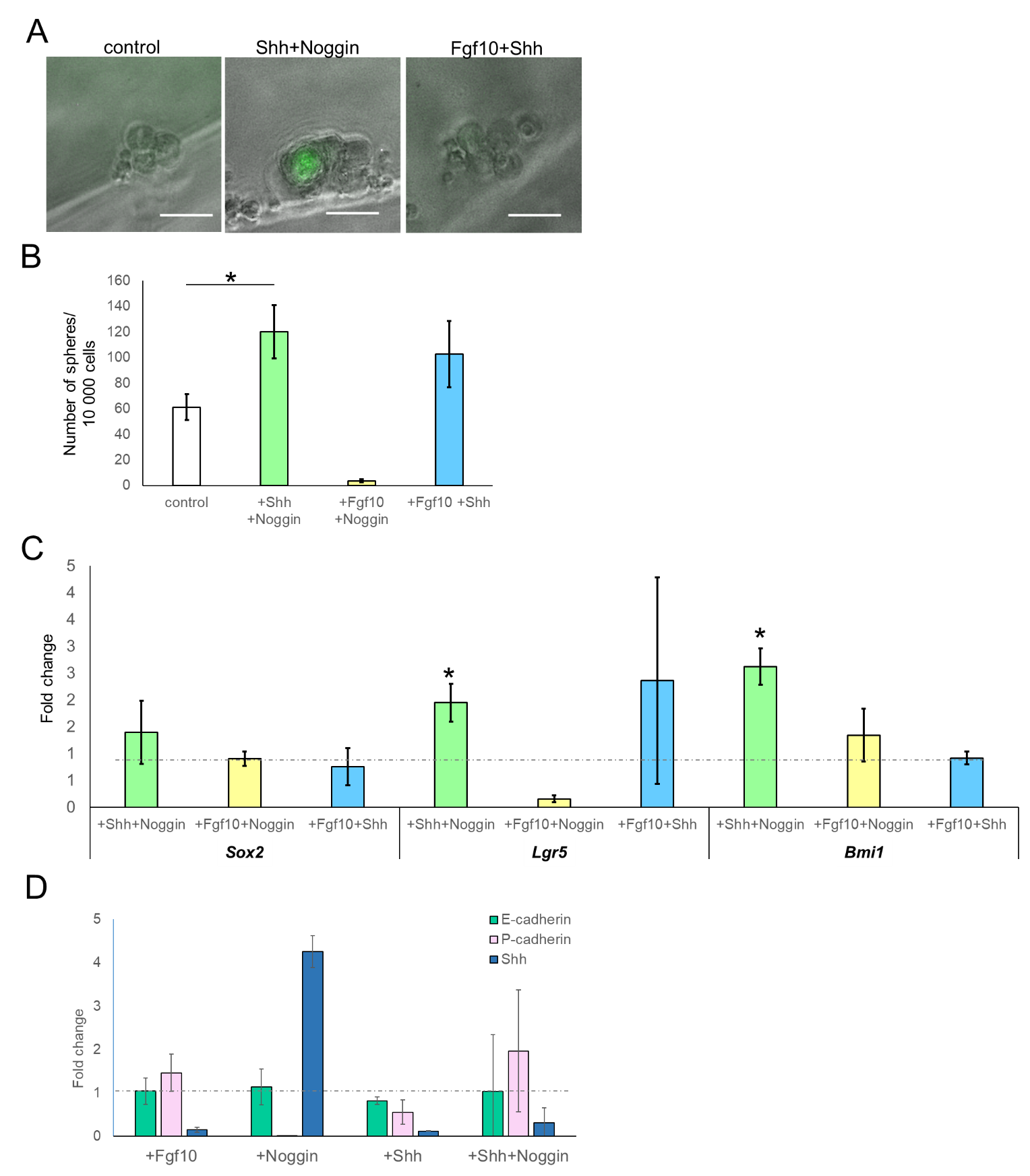


**Supplemental Figure 3. Analysis of the effect of protein combinations on Sox2-GFP+ cells and sphere formation. (**A) Sox2-GFP expression in control cultures and those treated with Shh/Noggin and Fgf10/Shh combinations. (B) Number of spheres formed in these cultures. N=3, *p value < 0.05 was determined by Student’s t-test. (C) RT-qPCR analysis of stem cell markers *Sox2, Lgr5,* and *Bmi1* in 14 days old cultures. N=3, *p value < 0.05 was determined by Student’s t-test. (D) RT-qPCR analysis of *E-cadherin, P-cadherin, and Shh* expression in 14 days old cultures.


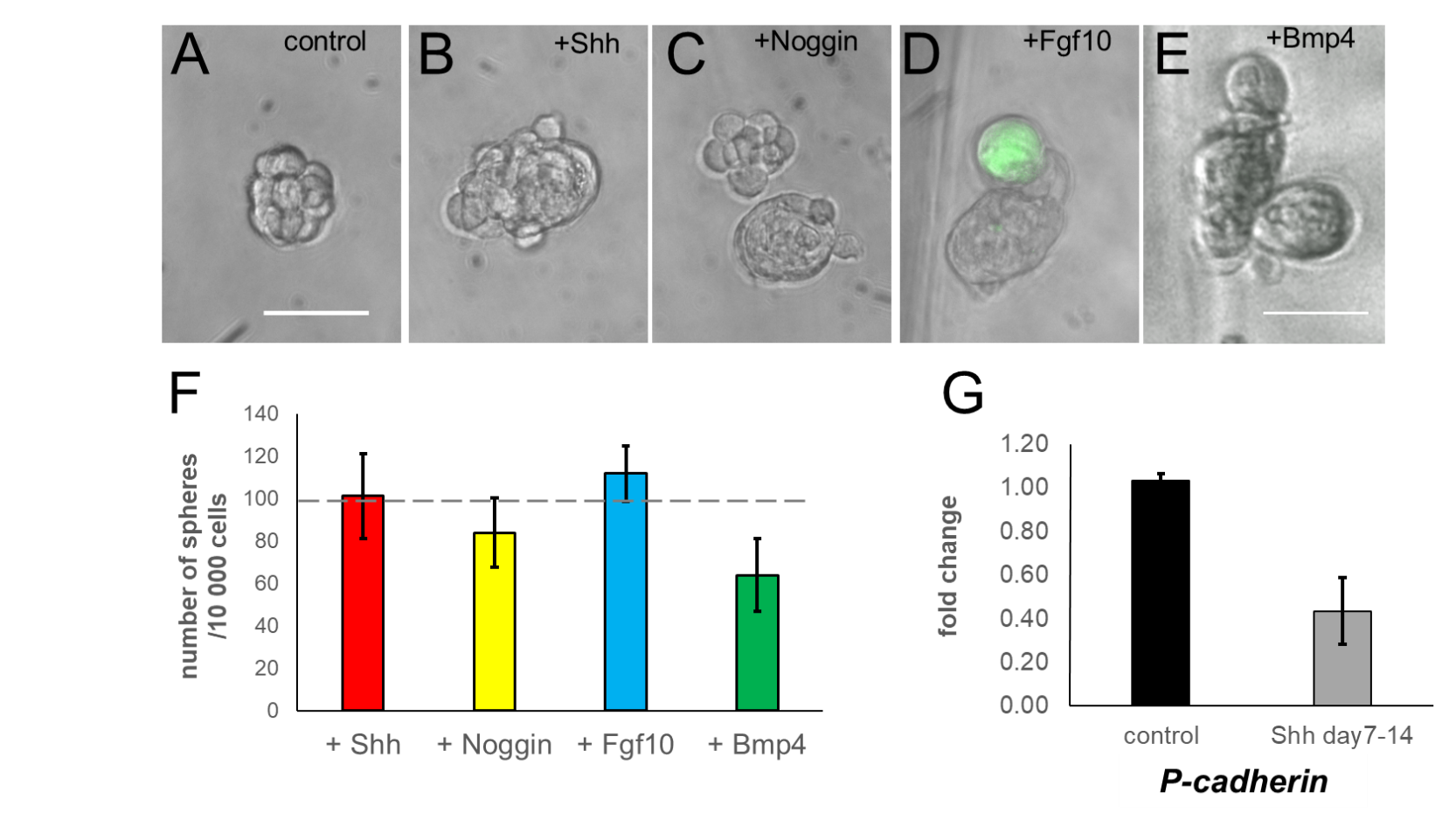
**Supplemental Figure 4. Effect of the growth factors and signalling molecules on the differentiation of Sox2+ stem cells.** (A-E) Analysis of Sox2-GFP expression in sphere culture treated with Shh, Noggin, Fgf10, or Bmp4 from day 7 until the end of culture. (F) Quantification of the sphere formation capacity (number of spheres formed per 10 000 cells). (G) RT-qPCR analysis of *P-cadherin* in 14 days old cultures treated with Shh from day 7 until the end of culture.


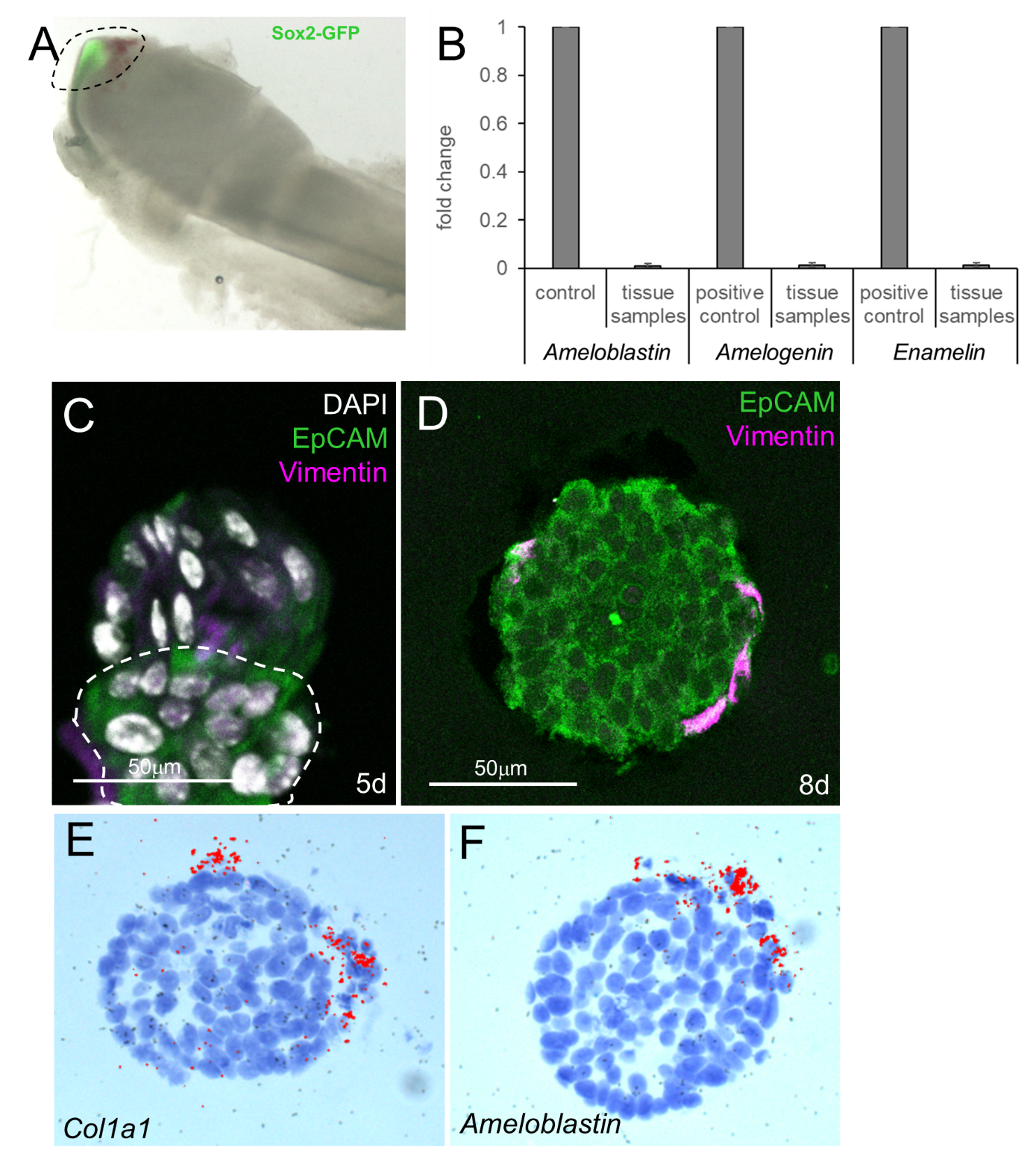


**Supplemental Figure 5. *In vitro* co-culture of epithelial and mesenchymal cells.** (A) Incisor tooth isolated from Sox2-GFP; Enamel-tdTomato transgenic animal and imaged for Sox2-GFP expression. Black dotted line indicates the portion of the tooth that was isolated and enzymatically dispersed. (B) qRT-PCR analysis of *Ameloblastin, Amelogenin* and *Enamelin* in the tissue portion obtained in A and comparisson to control (same tissue portion from A and ameloblast layer extending from the cervical loop). (C, D) Immunostaining against Vimentin (pink) and EpCAM (green) in sections of the 5 day old sphere, prior to transfer to Matrigel (C) and the spheres three days after the transfer to Matrigel (D). *In situ* hybridization for *Col1a1* (E) and *Ameloblastin* (F). Scale bar 50 μm.
